# Use of volunteers in early years interventions for parents: A scoping review of roles and the extent of evaluation research in this area

**DOI:** 10.1371/journal.pone.0305551

**Published:** 2024-09-26

**Authors:** Louise Padgett, Sarah L. Blower, Hollie Henderson, Maria Bryant

**Affiliations:** 1 Department of Health Sciences, University of York, York, United Kingdom; 2 Department of Health Sciences and the Hull York Medical School, University of York, York, United Kingdom; Curtin University Bentley Campus: Curtin University, AUSTRALIA

## Abstract

Early years interventions, particularly those supporting parents in the first 1001 days of their infants life, support healthy development of infants and prevent adverse experiences that can have long term negative impacts. Volunteers are often used to deliver such interventions. This scoping review aimed to explore the roles and characteristics of volunteers across early years interventions and map the evaluation in this field to identify gaps in the literature. A scoping review was conducted according to the Arksey and O’Malley Framework. Academic databases and grey literature sources were searched to identify literature evaluating volunteer-based interventions for parents in the first 1001 days of their infant’s life (conception to aged two). Research conducted in the UK or comparable high-income countries since the year 2000 were identified and data relating to the volunteer role, intervention design and evaluation methods were mapped. Sixty-six articles were eligible for inclusion in the review. Volunteers were commonly involved in interventions to provide peer support for a range of parenting related matters, support breastfeeding and the mental and emotional wellbeing of parents. Volunteer roles were categorised based on their background (peers or non-peer volunteers), their responsibilities (provider of peer support, educator or providers of practical support) and the delivery style of their role (in person one to one support, group leader or remote support). Research most often involved exploring the experiences of those involved in receiving or delivering the interventions and measuring outcomes in relation to breastfeeding and parent mental wellbeing. Volunteers play a large role in the provision of early years interventions. Their varied contribution is presented through a typology that will allow comparisons of roles in future research. Further research exploring the impact on the volunteers and the organisation will support decision making around choosing a volunteer led model within early years services.

## Introduction

The early years, and more specifically the first 1001 days of life (from conception to aged 2 years), is a crucial period in a child’s development [1] with adverse experiences (i.e., neglect and poor attachment with caregivers [2, 3]) during this period having the potential to cause negative impacts on health and prospects that last through to adulthood [4]. Interventions to support development and prevent adverse experiences remain a priority within UK government [5].

A range of universally offered and targeted early years interventions are provided to support parents in the early years [6, 7]. Early years interventions are provided to parents and their families in the early stages of their child’s life to support healthy development, which can prevent problems with a child’s health and prospects in later life [8]. One early years intervention delivery approach is to include volunteer roles within the delivery of the service [9]. The inclusion of volunteers across the broad spectrum of Health and Social Care Services has been suggested to generate benefits to the intervention recipients (i.e., improved wellbeing and reduced social isolation [10, 11]), the organisation hosting the service (i.e., increased reach of services through larger workforces [12]), the volunteers themselves (i.e., improved health and wellbeing [13]) and the wider community (i.e., improved social cohesion [12]) [11, 13–15]. However, some evaluations of volunteer led services have found limitations in relation to volunteer burnout and reduced sustainability of services [16, 17]. In addition, there is broad variation in the types of volunteer roles taken on across the different health services [11] which likely influences the impact the intervention can have.

Volunteering rates are highest within high-income countries such as United States of America (USA), Canada, Australia and United Kingdom (UK) [18], with volunteers making a substantial contribution within health and social care settings [10, 19]. Specifically within the UK, the third sector provides an increasing contribution of the delivery of public services such as early years interventions [20]. The third sector predominantly includes charities and non-profit organisations, and is where approximately two thirds of volunteers provide their contribution [21]. Within the Government’s vision for the 1001 critical days, the important role of this sector and volunteers is clearly highlighted [5], and it is likely that volunteers will continue to play an important role in the provision of early years interventions. The potential for volunteers to be involved in the delivery of early years interventions which can have a large influence on the healthy development of children has generated the need for research to be conducted to evaluate and explore the impact of early years interventions that include volunteer roles.

Reviews of early years interventions involving volunteers have been previously carried out in relation to specific outcomes [9] or intervention settings [22, 23] with findings guiding the implementation of volunteer roles within specific contexts. Previous reviews have been rapid reviews conducted to support future intervention design within their specific settings (i.e. Better Start services and Children’s Centres) [9, 22] and have therefore focused their literature searches on their specific areas of interest rather than looking at all early years interventions more broadly. These reviews consolidated findings from research using varied methods (i.e., quantitative and qualitative methods) and exploring different outcomes with only a small number of studies exploring each outcome [9, 22, 23]. In addition, previous reviews had different aims [22, 23] and therefore many of the included studies would not be relevant within this review specifically exploring interventions delivered in the first 1001 days. It was therefore unclear what the available evidence base was for volunteer led interventions for parents in the infants first 1001 days highlighting a need for a scoping review to map the available evidence. Further, the most recently published review was in 2016 and a more up to date review was warranted.

The purpose of this scoping review was to map the available evidence base of early years interventions delivered by volunteers. Mapping the literature was intended to identify where and how most evaluation has been conducted to date, the outcomes of interest to researchers when evaluating interventions involving volunteers and identify where further research of volunteers within early years interventions for parents may be needed. This was intended to provide guidance to professionals and researchers in the field of the available evidence base that could be used to determine if including volunteer roles within a particular early years intervention would be appropriate.

This review was also conducted in order to provide an understanding of the roles of volunteers within interventions for parents of infants within their first 1001 days and to develop a typology of the roles that volunteers take on across different interventions and services. This scoping review explored the most up to date literature in order to map different types of volunteer roles, the characteristics of volunteers and the purpose and designs of the interventions volunteers are involved in.

Scoping review methodology was applied as it suited the review’s purpose (i.e. to determine the scope and coverage of a specific topic and identify gaps in the research) rather than determining the effectiveness of these interventions which would have been suited to a systematic review. Mapping the literature and creating a typology of volunteer roles was intended to provide an understanding of the contribution of volunteers within early years interventions and help to distinguish and compare different types of volunteer roles within future research. In addition, providing an understanding of the range of volunteer roles across the different early years interventions will highlight the potential value of volunteers to those involved in the commissioning, design and delivery of early years interventions.

## Methods

A scoping review was conducted as this method enables the mapping of evidence in a particular field to understand more about a specific context (i.e. the role of volunteers in early years interventions) and identify where there are gaps in the literature [24]. The review was conducted according to the Arksey and O’Malley framework [25] which involved five key stages: identifying the research questions, identifying relevant studies, study selection, charting the data and collating, summarising, and reporting the results.

Results are reported according to the requirements of the PRISMA extension for scoping reviews (PRISMA-ScR) [26] (see S1 Checklist) and the protocol for the scoping review was pre-registered and published on the Open Science Framework (https://osf.io/yurj6).

### Identifying the research question

The research question of the scoping review was: *What are the roles of volunteers in interventions for parents of children in their first 1001 days and how are their roles evaluated*? The review objectives were: 1) To identify and create a typology of the different roles that volunteers play in the delivery of interventions for parents of children within their first 1001 days. 2) To explore the characteristics of volunteers within interventions for parents of children within their first 1001 days. 3) To explore the methods used to evaluate interventions for parents of children within their first 1001 days that include a volunteer role or to evaluate the volunteer role itself. 4) To explore the outcomes of interest reported within evaluations of interventions for parents of children within their first 1001 days that include a volunteer role. Outcomes included those relating to the services, service users and/ or their children and volunteers themselves.

### Identifying relevant studies

**Search strategy.** The search strategy was developed based on guidance from the Cochrane Collaboration [27] and Campbell Collaboration [28] and search strategies used within previous reviews relating to early years [9, 29] and volunteering [13, 30].

Search terms were based on the ‘Population’ in receipt of the intervention (i.e., parents and infants), the ‘Concept’ (i.e. volunteer roles), and the ‘Context’ (i.e., interventions and services) [31]. Additional terms relating to key outcomes and purposes of interventions were included to increase the specificity of search results. Search terms relating to each category of the strategy (i.e., population, concept, context and intervention outcome and purpose) were combined using the Boolean term OR and each category was then combined using the Boolean term AND (see S1 Text).

In order to identify the extent of the literature that could address the review objectives, both academic peer reviewed journals and grey literature sources were searched. The databases Medline, Psycinfo and Embase (Ovid), Applied Social Sciences Index & Abstracts (ASSIA), and Web of Science were searched using the prespecified search terms in May 2021 and updated in July 2023. The grey literature database HMIC (Health Management Information Consortium) was also searched. Search terms were identified within titles, abstracts, or key words. Mapping terms to subject headings (i.e. MeSH terms) was used within applicable databases to increase the efficiency and precision of the search [32]. Where possible, database searches were refined to documents published in English since the year 2000 and to exclude conference abstracts and student dissertations.

Web pages of organisations relevant to the review were hand searched for potentially eligible documents. Organisations included Early Intervention Foundation (EIF), National Institute for Health and Care Excellence (NICE), Department of Health, Nesta, Barnardo’s, Action for Children, Family Action and NSPCC. In addition, the internet search engine Google was searched using the same search terms through the advanced search functionality. The first five pages of results (based on 10 results per page) were screened as a minimum with an additional two pages being screened due to relevant searches continuing to be identified.

Reference lists of all eligible documents were screened to identify any eligible documents not identified through the search strategy.

### Study selection

#### Eligibility criteria

The eligibility criteria were developed based on the ‘Population’, ‘Concept’ and ‘Context’ of studies and criteria relating to the types of evidence source.

*Population*. The target population of interventions had to be parents and primary carers of infants within their first 1001 days (from pregnancy to two years of age). Interventions that also included parents of infants older than two years were eligible provided that the majority of children were aged under two (based on mean age or age range of sample). Interventions that also include the infants as intended beneficiaries along with the parent/ primary caregiver were eligible for inclusion.

*Concept*. Interventions had to include volunteer roles within some aspect of the delivery of the service. Volunteering was defined as “unpaid work conducted for the benefit of others beyond close relatives” [11] and therefore included any type of role (i.e., peer supporters and mentors) provided it was clearly stated they did not financially benefit from their role. Interventions where volunteers received expenses were eligible.

*Context*. Interventions conducted in the UK and comparable high income countries (based on the World Bank List 2020/ 2021 https://datahelpdesk.worldbank.org/knowledgebase/articles/906519-world-bank-country-and-lending-groups) where volunteering rates are highest [18] and where healthcare systems are similar [33] were eligible. Eligible interventions were those that were designed to support parents during the transition to parenthood and up to an infant being aged two years, including those to support parenting (skills, confidence or satisfaction), parent wellbeing (emotional, mental or social), breastfeeding or to support the attachment and parent-child relationship.

*Evidence source*. Eligible evidence sources were required to provide details of the intervention purpose and delivery style, volunteer role and outline the purpose and methods used in evaluating the intervention or exploring the volunteer role. Studies using quantitative, qualitative and mixed methods were eligible for inclusion. Eligible documents were required to be available in the English language and published from the year 2000 onwards. Reviews, protocols, conference abstracts, dissertations and letters to editors were not eligible for inclusion to prioritise documents most likely to include all data necessary to address the review objectives. The decision to focus on documents published since the year 2000 was based on the rapid review conducted by McLeish, Baker [9] which explored early years interventions involving volunteer roles relating to the “A Better Start” programme outcomes which focused on studies published from 1990 to 2015. The reference list in this paper identified that few studies conducted in the 1990s were of relevance to this review and therefore this review focused on studies conducted from 2000. Due to differing objectives of this review compared to the review by McLeish, Baker [9] it was still deemed relevant to include eligible studies also identified by McLeish, Baker [9] from the year 2000.

#### Evidence selection

Retrieved documents were exported into the reference management software, Endnote [34], where duplicates were removed. Documents were then screened according to the eligibility criteria, firstly based on the title and abstract and then those deemed potentially eligible screened against the full text. Eligibility screening was conducted using the software, Rayyan [35]. To ensure the eligibility criteria was being applied correctly and consistently, at both title and abstract and full text screening stages, two reviewers (LP and HH) independently reviewed the first 10% of documents. Inter-rater reliability was assessed using Cohens Kappa score and a strong agreement score of 0.8 or above [36] was required before the remaining documents were screened by the first reviewer (LP) only. At full text screening stage, an additional 10% of documents were reviewed due to a strong kappa score not being achieved after the first 10% of documents.

### Charting the data

#### Data extraction

Data relevant to the review objectives were extracted onto a piloted Google Form. The first 10% of documents were extracted independently by two reviewers (LP and HH) to ensure data was being extracted correctly and the remaining documents were then extracted by one reviewer (LP). Extracted data included author details, year and country of publication, intervention details such as target population, purpose, setting and delivery style of intervention, the role and characteristics of volunteers, methods used in evaluating the intervention or exploring the volunteer role and outcomes of interest within the evaluations.

#### Collating, summarising and reporting the results

Characteristics (i.e. evidence source data and population characteristics) and data relating to the review objectives (i.e. volunteer characteristics, intervention and study design) of each eligible source of evidence were collated and presented in a table. A narrative summary was conducted to provide an overview of evidence identified. This involved identifying and quantifying the different research methods and outcomes explored to describe and summarise the evidence base, and identify gaps in the evidence of early years interventions involving volunteers. Similarly, data describing volunteer roles and characteristics were identified and quantified to identify commonalities to categorise roles and form a typology. The purpose and design of early years interventions was summarised and mapped against volunteer role typologies to gain an overview of how and where volunteers are contributing across different early years interventions.

## Results

### Search results

The searching of all databases resulted in a total of 15,046 documents being identified. Following the removal of duplicates, 7,883 documents were screened against the eligibility criteria based on their titles and abstracts. 334 documents were deemed potentially eligible and were then assessed for eligibility based on their full texts. An additional 62 documents were identified though searching the Google search engine and organisation web pages and through citation searches and were assessed for eligibility.

**Fig 1:**
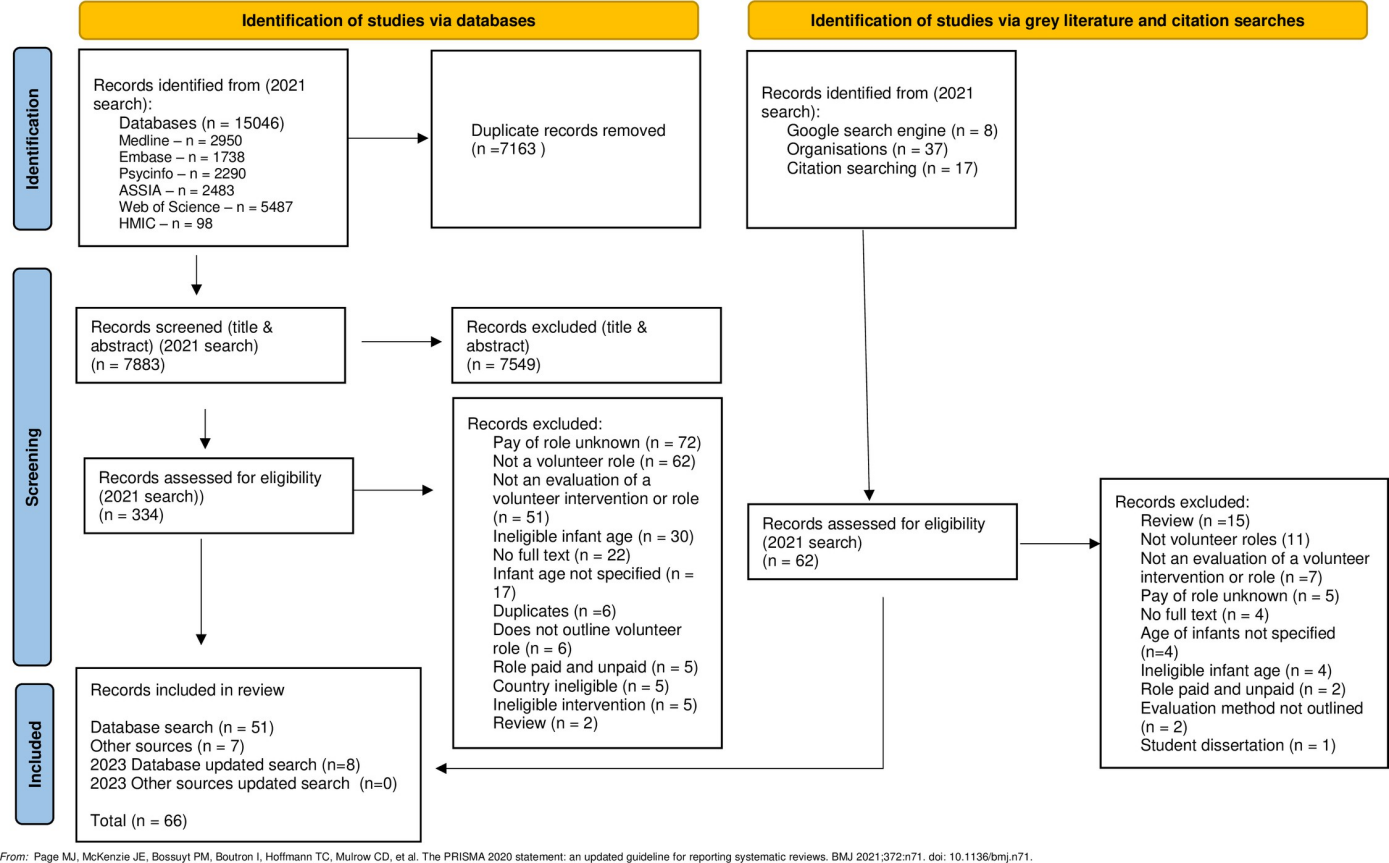
Flow diagram of included studies.

**Fig 2:**
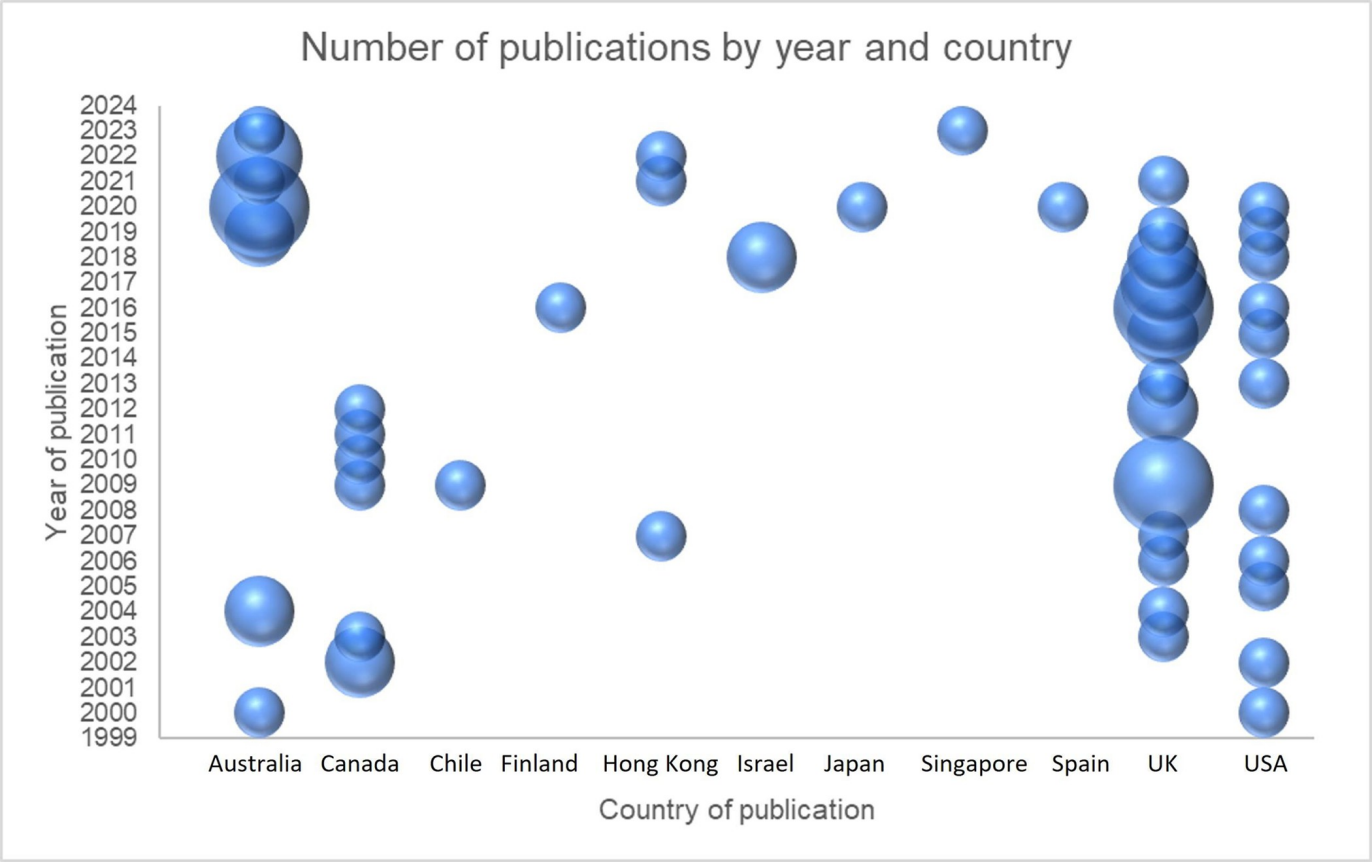
Bubble plot of publication date and country of included studies.

A total of 58 documents were deemed eligible for inclusion in the review, 51 of which were identified from databases and 7 from grey literature sources. Database and grey literature searches were updated in 2023 which resulted in an additional 8 eligible documents. Over half of publications were published since the year 2015 (n = 37) with most published in the UK (n = 24), Australia (n = 14), USA (n = 11) and Canada (n = 7). Fig 1 provides details of the flow of documents and reasons for exclusion of documents at the full text screening stage and Fig 2 presents the number of publications by year and by country.

The final 66 documents included evaluations of 55 different interventions. Six of the 55 interventions had multiple evaluations reported over multiple documents which accounts for there being more documents than individual interventions.

### Characteristics of included articles and evidence base

Details of included study methods and outcomes can be seen in Table 1. Where intervention details and details of volunteer roles have been combined, this is to demonstrate that there have been multiple articles reporting different evaluations of the same intervention.

**Table 1:** Study characteristics.

Lead author, date and country	Intervention details			Details of Volunteer role			Evaluation details		
	Target population and recruitment approach	Intervention name (if available) & Intervention Purpose	Delivery style and duration	Title and background	Capacity of role	Training	Evaluation purpose, method	Participant/ perspective of interest	Outcomes/ phenomena of interest
Aracena 2009 [102] Chile	Adolescent mothers recruited via Public Health Register from pregnancy to infant aged 1 year.	Teaching parenting skills, helping to create mothers identity and make future plans	Home visiting 12 x 1 hour sessions	Volunteer, Health educator	Educate	Trained, amount not specified	Intervention effectiveness and cost effectiveness Randomised Controlled Trial	Mother and infant	Mother physical health, mental health, family function, child’s physical health, psychomotor skill, indicators for child abuse post intervention. Cost measurement and cost effectiveness
Barnes 2009 [38] UK	Mothers during pregnancy that score above 9 on social disadvantage index, living outside a sure start area recruited from waiting areas of antenatal clinics from pregnancy to infant aged 1 year.	**Home Start** Teaching parenting skills, practical support (i.e. childcare, housework) based on parent need	Home visiting 15 visits over an average of 5.5 months	Volunteer Parent and local community member	Provide practical support and advice dependent on needs of mothers	3.5 days	Intervention effectiveness Randomised Controlled Trial	Mother	Primary outcome: major or minor depression at 2 and 12 months Additional outcomes: depression symptoms at 12 months.
Macpherson 2010 [69] UK							Descriptive Qualitative interviews	Mother	Issues relating to and perceptions of receiving support at 2 and 12 months.
Barnet 2002 [83] USA	Young mothers only between 28 weeks gestation and 6 months recruited through schools.	Teaching parenting skills, signposting to services and support parent to continue education	Home visiting 1.5 hours per week for up to a year	Volunteer Local community member	Provide emotional support, provide peer support, educate and signpost to services	16 hours	Intervention effectiveness Randomised Controlled Trial and structured interviews	Mother	Main outcome measures: Parenting stress, parenting behaviours and mental health at 15 months.
Bhavanni 2016 [58] UK	Mothers during pregnancy and early years recruited via self-referral.	**Shared Experiences Helpline** Peer support to reduce parent concerns around child wellbeing.	Telephone support As and when required by service user	Volunteer, Peer supporter Parent of an infant who has had difficult experiences	Provide peer support	1 day	Descriptive Qualitative interviews	Mother	How telephone peer support was experienced
Bogat 2008 [59] USA	Adolescent pregnant mothers recruited through school.	Support mental and emotional wellbeing, teaching parenting skills, and mentoring	One to one 2 hours per week for an average of 6.8 months	Mentor Mothers of an infant recruited through advertising	Provide emotional support, educate, provide practical support and companionship	4 hours	Exploration of mentor mentee relationship Qualitative analysis of supervisor notes and mentor logbooks	Mother and volunteer	Mentor and mentee experiences (e.g., thoughts, attitudes, and behaviours) in the mentoring relationship
Burns 2020 [84] Australia	Mothers of infants aged 1 week—12 months recruited via self-referral or clinical referral.	**Australian Breastfeeding Association peer counselling** Breastfeeding education and support and peer support	One to one, and group sessions in the community. As and when required by service user.	Volunteer peer support counsellors Specifically trained counsellors	One to one breastfeeding consultations	900 hours	Descriptive Quantitative and qualitative questionnaire and surveys	Mother	Experience of drop-in service
Cattelona 2015 [60] USA	Mothers during birth and postpartum period. Recruitment method not reported.	**Bloomington Area Birth Services** Breastfeeding education, peer support and birth support	One to one at home or clinical setting. A minimum of 3 visits.	Doula	Provide emotional support and educate	Trained, amount not specified	Descriptive Qualitative case study	Mother	Mothers experience of doula support and their breastfeeding outcome
Coe 2013 [85] UK	Mothers from pregnancy to 1 year with mild to moderate mental health issues via self-referral or clinical referral.	**The Perinatal Support Project** Support mental and emotional wellbeing, support attachment, reduce isolation and improve confidence	Group sessions in the community and home visiting. Duration not reported.	Volunteer befrienders	Befriend, provide emotional support and educate	6 days	Pilot study and service evaluation Pre post data collection and qualitative interviews	Mother, volunteer, project coordinator and referring agency staff	Service evaluation—mother anxiety and depression; social support; and self-esteem and volunteer self-esteem post intervention Stakeholder interviews—project coordinators, service users, volunteers and referring agencies perceptions of intervention
Curtis 2007 [61] UK	Mothers of infants of breastfeeding age in low-income areas recruited via clinical referral or through volunteers’ current social circles.	**Breastfriends Doncaster** Breastfeeding education and peer support	One to one in clinical settings and heath promotion within volunteers’ social circles. Duration not reported.	Volunteer, Peer supporter Young mothers from local low-income areas with breastfeeding experience	Provide peer support and educate	20 hours	Descriptive Qualitative focus groups	Volunteers and health professionals	Key elements of the peer-professional interface
Cwikel 2018 [86] Israel	Mothers from an area of high proportion of immigrants that have an infant up to 1 year recruited via clinical referral.	**Mom to Mom (M2M)** Support mental and emotional wellbeing and role modelling	Home visiting Weekly for up to a year	Volunteer Mothers	Provide emotional support, role model and educate	Not reported	Impact of intervention Quantitative and qualitative questionnaire with open and closed questions	Mother	Motivations for joining, reported gain and impact on postpartum depression at 1 year
Dennis 2002 [42] Canada	Mothers recruited at birth of infant recruited from hospital wards.	Breastfeeding education and peer support	Telephone support 12 weeks as and when required by service user	Volunteer, Peer Minimum 6 months breastfeeding experience	Provide peer support	2.5 hours	Intervention effectiveness Randomised Controlled Trial	Mother	Primary outcome: post-natal depression at 12 and 24 weeks Additional outcomes: anxiety, loneliness and health service utilisation at 12 and 24 weeks
Dennis 2002b [63] Canada							Descriptive Questionnaire, qualitative interviews and volunteer activity logs	Mother, Volunteer	Perceptions of peer support at 12 weeks Peer volunteer experience and nature and intensity of interventions
Dennis 2010 [41] Canada	Mothers recruited at 2 weeks postpartum with an Edinburgh Postnatal Depression Scale score >9 recruited via standard postpartum care calls.	Peer support	Telephone support 12 weeks with a minimum of four contacts, then based on need of service user	Peer Mothers who have recovered from Postnatal Depression	Provide peer support	4 hours	Acceptability and descriptive Quantitative questionnaire	Mother	Maternal perceptions of support at 12 weeks.
Dennis 2003 [39] Canada	Mothers of infants aged 8–12 weeks, at risk of Postpartum depression, defined by a score of above 9 on Edinburgh Postnatal Depression Score recruited through infant immunization appointments.	Support mental and emotional wellbeing, social support and appraisal	Telephone support As and when required by service user	Peer volunteers Mothers with previous experience of a mental health condition	Provide emotional support, provide peer support	4 hours	Pilot study and intervention effectiveness Randomised Controlled Trial	Mother, Volunteer	Primary outcome: depressive symptomology at 8 weeks Additional outcomes: maternal self-esteem, childcare stress, maternal loneliness, maternal perception of peer support, peer-volunteer perceptions of peer support, peer-volunteer activities.
Dennis 2009 [40] Canada	Mothers of infants aged 2 weeks and at risk of postpartum depression recruited via clinical referral.	Peer support, signposting to services and prevent postpartum depression	Telephone support 10 weeks	Peer volunteer Mothers	Role model, peer support	4 hour training	Intervention effectiveness Randomised Controlled Trial	Mother	Primary outcome: Postnatal depression at 12 and 24 weeks
Downie 2004 [64] Australia	First time parents of infants aged 0 to 1 year. Recruitment method not reported.	**Community Mothers** Support parenting confidence and child development	Home visiting 1 year	Volunteer, Community mothers Local mothers	Role model	Trained, amount not specified	Descriptive Qualitative interviews	Volunteer	Perceptions and experiences of volunteers. Reasons for becoming a volunteer and exploration of their role
Fan 2022 [55] Hong Kong	Women with intention to breastfeed recruited at antenatal classes in a public hospital	Increase breastfeeding rates.	Text based phone support. Ad hoc.	Peer Counsellors Women with at least 2 months breastfeeding experience and with training.	Answer breastfeeding related questions and provide emotional support.	Training amount not specified	Feasibility and acceptability Pilot Randomised Controlled Trial	Mother	Feasibility assessed by proportion of women who agree to participate and be followed up. Acceptability assessed by perceived helpfulness of intervention. Exclusive breastfeeding at 1,2,4 and 6 months.
Forster 2019 [43] Australia	First time mothers planning on breastfeeding recruited at birth of infant recruited at hospitals.	Increase breastfeeding at 6 months	Telephone support Weekly for 12 weeks	Peer volunteers Mothers who have breastfed until 6 months	Provide emotional support, provide peer support and signpost to services	4 hours	Intervention effectiveness Randomised Controlled Trial	Mother	Primary outcome: proportion of infants breastfeeding at 6 months Additional outcomes: exclusive breastfeeding and time to cessation
Grimes 2020 [66] Australia							Descriptive Qualitative focus groups analysed based on volunteerism theory framework	Volunteer	Issues thought to impact the volunteers that may impact on duration of participation and to elucidate their personal experience of providing support.
McLardie-Hore 2020 [92] Australia							Intervention evaluation Quantitative and qualitative questionnaire	Mother	Satisfaction with service at 6 months
Grimes 2021 [80] Australia							Implementation of intervention Data collected form volunteers throughout recruitment and training and from call logs.	Volunteers and volunteer coordinator	key aspects of recruitment, training and support of the peer volunteers details regarding the key topic areas discussed during the calls as well as referrals suggested by volunteers. Volunteers’ perceptions of the value of the calls to mothers; details regarding the role of the peer volunteer coordinator.
McLardie-Hore 2022 [81] Australia							Descriptive Qualitative in-depth interviews	Mothers	Women’s experience of receiving peer support.
McLardie-Hore 2023 [57] Australia							Cost effectiveness	NA	Costs within 6 month follow up period relating to individual healthcare, breastfeeding support, intervention costs. Incremental cost-effectiveness ratio.
Gonzalez-Darias 2020 [44] Spain	First time mothers recruited at birth of infant recruited from hospital.	**Supporting a First Time Mother** Peer support and increase breastfeeding rates	Online As and when needed by service user for 6 months	Peer supporter Mothers who have exclusively breastfed until 6 months	Provide peer support and educate	4 hours	Intervention effectiveness Randomised Controlled Trial	Mother	Breastfeeding rates at 3 and 6 months
Graffy 2004 [45] UK	Mothers during pregnancy (28 to 36 weeks gestation) recruited from areas with varying levels of deprivation recruited via antenatal care at GPs.	Breastfeeding education and counselling	Home visiting and telephone support. Once before birth then as and when needed by service user.	Volunteer counsellors Mothers with breastfeeding experience	Provide peer support and breastfeeding support	Trained, amount not specified	Intervention effectiveness Randomised Controlled Trial	Mother	Primary outcome: prevalence of any breastfeeding at 6 weeks Additional outcomes: the proportion of women giving any breast feeds, or bottle feeds at four months, the duration of any breast feeding, and time to introduction of bottle feeds.
Granville 2012 [65] UK	Vulnerable parents during pregnancy to infant being aged 3 months recruited via self-referral or organisation referral.	**Parents 1**^**st**^**. Pregnancy pals and Birth Buddies** Peer support, support positive births and best start to life	One to one at home and group sessions in the community. Until infant is aged 3 months.	Volunteer, Peer	Deliver class and provide peer support	Trained, amount not specified	Descriptive and evaluation of intervention impact Qualitative interviews and focus groups analysed based on theory of change	Mother, volunteers, midwives, counsellors and social workers	The outcomes tested in this evaluation were: 1. Families are confidently prepared for birth and parenthood 2. Parents with young children have built confidence and parenting skills 3. Parent volunteers: Accredited training leads to employment pathways and community involvement 4. Parents, volunteers and children have improved health and wellbeing
Gruber 2013 [46] USA	At risk mothers recruited at birth recruited via clinical referral, organisation referral or recruited from a parent education class.	**YWCA Grensboro Healthy Beginnings** Support mental and emotional wellbeing and breastfeeding education	One to one and group sessions in the community. Duration not reported.	Doula Mixture of professional and nonprofessional backgrounds	Provide emotional support and educate	Trained, amount not specified	Intervention impact Nonexperimental design, using routinely collected comparative group data	Mother	Type of birth, incidence of having a Low Birth Weight baby, incidence of complications at birth for either the mother or baby, and incidence of initial breastfeeding.
Hiatt 2000 [47] USA	First time mothers from birth to infant being aged 1 year who are deemed low risk recruited via clinical referral or organisation referral.	**Kempe Community Caring Program** Teaching parenting skills and befriending	Home visiting and telephone support. 2 visits and 3 phone calls up to infant being aged 1 year.	Volunteer Varied backgrounds	Befriend and educate	4 hours then monthly 2 hour educational meetings	Intervention evaluation Quantitative pre post data collection and questionnaire	Mothers and volunteer	Volunteer self-evaluation, support, satisfaction. Parent evaluation of perceived support and family functioning
Hongo 2020 [48] Japan	Mothers who have expressed willingness to breastfeeding recruited from hospitals.	Peer support, signposting to services and breastfeeding support	Telephone support. As and when needed by service user for 4 months.	Peer supporter Mothers with a minimum of 6 months breastfeeding experience	Provide peer support	2 days	Intervention impact Quantitative questionnaire	Mother	Breastfeeding satisfaction at 1 and 4 month postpartum
Hopper 2016 [67] UK	Mothers recruited at birth of infant. Recruitment method not reported.	Breastfeeding education and peer support	One to one in a clinical setting. Duration not reported.	Peer supporter	Provide peer support	Not reported	Descriptive Qualitative interviews	Volunteers and ward staff	Breastfeeding peer supporters’ motivation to volunteer within a hospital environment, their experiences of volunteering within a hospital environment, the relationships between peer supporters and ward staff, and to identify factors contributing to the future sustainability of the service.
Kaitz 2018 [87] Israel	Mothers from pregnancy to infant being age 1 year recruited via self-referral or professional referral.	**Mom to Mom (M2M)** Support mental and emotional wellbeing and improve infant care	Home visiting Weekly until infant is aged 1 year	Volunteer, Home visitors Local mothers	Provide peer support and caring for the baby	8 hours	Intervention evaluation Quantitative and qualitative questionnaire	Mother	Improved wellbeing and improved infant care at end of intervention.
Kane Low 2006 [68] USA	At risk mothers during pregnancy and postpartum. Recruitment method not reported.	**The Doulas Care Program** Teaching parenting skills, breastfeeding education and practical support	One to one at home Duration not reported	Doula	Support at appointments, parenting education and breastfeeding support	Trained, amount not specified	Descriptive Qualitative focus groups	Volunteer	Educational needs to overcome barriers to being a community doula
Kelleher 2004 [49] Australia	Mothers only with infants less than 6 weeks of age, vulnerable based on Parenting Readiness Checklist. Recruitment method not reported.	**Cottage Community Care Pilot Project** Teaching parenting skills, reduce isolation and prevent child maltreatment	One to one at home Weekly for an average of 8 months	Volunteer Diverse backgrounds i.e., nuns, mothers, teachers	Provide emotional support, educate, and signpost to services	20 hours	Descriptive Quantitative comparative pre post data collection	Mother	Mother child relationship (social supports, family conflict, stress and coping, self-esteem, confidence as a parent, stability and meeting basic needs, expectations, affective relationships, and sensitivity to caretaking functions) at 1 year or exit of intervention.
Kuliukas 2019 [88] Australia	Fathers recruited during pregnancy recruited via antenatal classes.	**Father-focused breastfeeding antenatal classes (FFAB)** Support fathers to be breastfeeding supporters	Group sessions in a clinical setting. 50 minutes one off session	Peer facilitator Mothers	Deliver class, provide peer support and educate	4 hours	Process evaluation Quantitative and qualitative questionnaire and qualitative interviews	Fathers and volunteer	Satisfaction of fathers post intervention Expectations and confidence to manage breastfeeding problems post intervention. Motivations and expectations of volunteers.
Law 2020 [50] Australia	First time mothers during pregnancy that are in a relationship and low risk of Postnatal depression recruited via public advertisement.	**The Mummy Buddy Programme** Support transition to motherhood	One to one at home. As and when needed by service user for 6 months.	Volunteer, Peer, Mummy Buddies Local mothers	Support plans for transition to motherhood and practical bespoke support	3 hour workshop	Feasibility and preliminary efficacy Quantitative pre post data collection and questionnaire.	Mothers and volunteer	Feasibility post workshop and preliminary efficacy based on depression symptoms at 12 and 24 week postpartum
Lederer 2009 [89] UK	Parents during pregnancy to infant being age 1 year and are at risk due to being a young mother, have existing or are vulnerable to mental health conditions and/ or are a refugee/ asylum seeker recruited via clinical referral or organisation referral.	**Perinatal Support Project (Family Action)** Support mental and emotional wellbeing, signposting to services, support attachment and engage families in wider community	Home visiting. 2 hours a week from 16^th^ week of pregnancy to infant being 1 year old.	Volunteer befrienders	Provide emotional support, educate, and support attachment	Trained, amount not specified	Descriptive and service evaluation Routine data, questionnaire/ survey and qualitative interviews	Parents, volunteers and project coordinators/ managers	Anxiety and depression, social support, the number, age, sex, ethnic grouping and mental health of both referrals and users of the project The number, age, sex and ethnic grouping of befrienders trained. The number of contacts through home visits. The number of contacts through drop-in groups. The number and nature of workshops or semi structured groups Experiences of service users, volunteers and project coordinators
Letourneau 2011 [51] Canada	Mothers of infants aged under 9 months with an Edinburgh Postnatal Depression Scale score above 9 recruited via clinical referral or through advertising.	Support mental and emotional wellbeing, peer support, support attachment and practical support	Home visiting On average 9 sessions over 12 weeks.	Peer Mothers of infants that have experienced Postnatal depression	Provide emotional support, provide peer support, role model, educate and signpost to services	8 hours	Intervention effectiveness Randomised Controlled Trial	Mother	Primary outcome: mother infant interactions at 6 and 12 weeks Additional outcomes: Depression symptomology, cognitive development, maternal report of social-emotional development, perceptions of social support, diurnal cortisol rhythm, intervention dose and content at 6 and 12 weeks
Lok 2021 [90] Hong Kong	Mothers intending to breastfeed recruited from hospital.	Signposting to services and promote and sustain exclusive breastfeeding	Home visiting. 5 visits over a minimum of 6 months.	Peer supporter Mothers with a minimum of 4 months breastfeeding	Signpost to services and breastfeeding support	Trained, amount not specified	Intervention feasibility and acceptability Randomised Controlled Trial, questionnaire and qualitative interviews	Mother	Feasibility and acceptability at one, two, four and six months Efficacy—breastfeeding status and breastfeeding self-efficacy at 2 months
Martin 2020 [91] USA	Mothers at birth of infant recruited via hospital.	**NurturePA** Support mental and emotional wellbeing and signposting to services	Text message support Duration not reported.	Mentor	Provide emotional support and signpost to services	3 hours	Feasibility Quantitative and qualitative analysis of text message scripts and program data	Mothers and volunteer	Feasibility at 1 year of enrolment
McLeish 2015 [70] UK	Vulnerable mothers (including young mothers, at risk, socially isolated, first time parent, with HIV or mental health conditions) with no support at birth recruited two weeks before birth recruited via self-referral, clinical referral or organisation referral.	Multiple interventions including peer support, supporting birth signposting to services and practical support	Group sessions, home visiting and telephone support. Duration varied across different settings (range from pregnancy to 2 years).	Volunteer, peer supporter, Doula Local mothers with previous experience of breastfeeding and/ or mental health condition	Peer support before, during and after birth and signposting to services	8 hours to 72 hours	Descriptive Qualitative interviews according to phenomenology	Mothers and volunteer	Mothers’ and volunteers’ perceptions of peer support across different interventions and peer support models
McLeish 2016 [71] UK	Mothers recruited during pregnancy and early motherhood living with HIV recruited via clinical referral.	Peer mentoring support	One to one at home and telephone support. As and when needed by service user.	Mother mentors Mothers living with HIV	Provide peer support	36 hours	Descriptive Qualitative interviews according to phenomenology	Mothers and volunteer	To explore the experiences of women living with HIV in England who received or gave Mentor Mother (trained mother-to-mother) volunteer peer support during pregnancy and early motherhood.
McLeish 2017 [72] UK	Mothers during pregnancy to infant being two years of age including young mothers, at risk mothers, insecure migrant status, experience of substance abuse or crime and/ or mental health conditions. Recruitment method not reported.	Various services providing support to women during pregnancy and first two years of infants life	Home visiting and telephone support. Duration varied by intervention (range from pregnancy to 2 years).	Volunteer Local mothers	Range of services providing support to women during and after pregnancy	ranging from 8–75 hours	Descriptive Qualitative interviews analysed according to phenomenology, underpinned by contextualism	Volunteer	Understand the volunteers’ experiences of supporting very disadvantaged mothers.
McLeish 2018 [73] UK	Mothers only. At risk, socially isolated, no partner, poor mental health or domestic violence or recent migration. Recruited via self-referral or clinical referral.	**3 x Community Doula Projects** Provide social support during and after pregnancy	One to one at home and telephone support. Weekly	Doula Local community member with specific Doula training	Provide emotional support	75 hours	Descriptive Qualitative interviews analysed according to phenomenology and social psychology	Mothers and volunteer	Understanding and experience of the community doula role during labour and birth
McLeish 2019 [74] UK							Descriptive Qualitative interviews analysed according to phenomenology.	Mothers and volunteer	Motivation for volunteering, training, activities as a doula, support received from the project, experience of working alongside health and social care professionals, and impacts on supported women and on the volunteer Mother’s experience of using the maternity services, the support they had received from the doula, the impact of the doula support, and their opinion of the doula support.
McLeish 2021 [82] UK	Disadvantaged mothers. The nine different projects had “a variety of target groups including young mothers, South Asian mothers, refugee and asylum seeker mothers, mothers living with HIV, mothers with mental health difficulties, mothers with very complex needs or any disadvantaged mother in the local area.”	9 different projects that provide social support to disadvantaged mothers.	One to one and group support. During pregnancy and until the baby was between 6 weeks and two years old.	Volunteer Mothers with peer experiences	Provide social support. Active listening, providing information and signposting to local services.	Training amount not specified	Descriptive Qualitative semi structured interviews. Informed by the theoretical perspective of phenomenological social psychology.	Mothers	Experiences of disadvantaged mothers who received social support from volunteers.
Mugweni 2018 [93] UK	Parents during pregnancy to infant aged 1 year who live in a deprived locality. Recruitment method not reported.	**Medway Perinatal Support Project (Family Action)** Support mental and emotional wellbeing, support attachment and improve outcomes of child	Group sessions in the community and home visiting. Up to infant being 1 year of age.	Volunteer befriender Parents who are local community member	Befriend and provide emotional support	6 weeks	Service evaluation Quantitative pre post data collection, and qualitative interviews and focus groups based on theory of change	Parents (both), volunteers and partner agencies	Maternal mental health, self reported anxiety and depression. Parent/ infant relationship Perceptions of experiences
Muirhead 2006 [94] UK	Mothers from birth of infant to infant aged 16 weeks recruited from hospital.	Breastfeeding education and peer support	Home visiting and telephone support. Every 2 days for first 28 days then as and when needed by service user up to 4 months.	Peer supporter Mothers with breastfeeding experience	Provide peer support	2 days	Intervention effectiveness Randomised Controlled Trial and qualitative questionnaire	Mother	Primary outcome: differences in breastfeeding initiation and duration at 10 days, 8 weeks and 16 weeks Qualitative data on problems encountered, solutions and types of support
Neel 2019 [75] USA	Mothers during pregnancy to postpartum. Recruitment method not reported.	Support mental and emotional wellbeing and support birth	One to one in a clinical setting. Duration not reported.	Doula Women with specific training	Provide emotional support	Trained, amount not specified	Descriptive Qualitative interviews	Health professionals	Best practices for integrating doulas into hospital‐based maternity care teams
Niela-Vilen 2016 [52] Finland	Mothers recruited at birth of infant. Recruitment method not reported.	Breastfeeding peer support	Facebook group. Duration not reported.	Volunteer, Voluntary mothers Mothers with breastfeeding experience	Provide peer support	No training provided	Feasibility and intervention impact Randomised Controlled Trial and quantitative questionnaire	Mother	Primary outcome: breastfeeding duration at 3 months, 6 months and 1 year Additional outcomes: Breastfeeding attitude and breastfeeding self-efficacy Intervention feasibility at 3 months, 6 months and 1 year
O’Rourke 2022 [101] Australia	Women experiencing socioeconomic adversity (financial hardship, refugee background, homelessness or complex trauma).	Provide social, emotional and practical support.	Home visiting and support in clinical settings. During labour and two post natal visits.	Volunteer doula Private doula background, midwives and midwifery students and bicultural workers.	Provide social, emotional and practical support.	Training amount not specified	Realist evaluation Interviews, focus groups and routine collected data	Mothers and volunteers	How, when and why the community volunteer doula programme works in improving outcomes for women.
O’Rourke 2022 [100] Australia							Realist evaluation Interviews, focus groups and routine collected data	Mothers and volunteers	Test and refine initial theories about cultural matching being best for women.
Paris 2005 [76] USA	Mothers recruited at birth who are at risk, socially isolated or at risk of depression. Recruitment method not reported.	**Visiting Moms** Support mental and emotional wellbeing, teaching parenting skills and practical support	Home visiting. Weekly for up to 1 year.	Volunteer Local experienced mothers	Provide emotional support, provide peer support, role model and educate	Trained, amount not specified	Descriptive Qualitative interviews analysed through the lens of Relational Cultural Theory	Mother	The relationship that developed with their home-visitor and its impact on their lives at 6–8 months in to intervention.
Prevatt 2018 [103] USA	New mothers of infants on average 18 months old recruited via self-referral or clinical referral.	Support mental and emotional wellbeing, Peer support and prevent Postpartum mood disorders	Group sessions in a clinical setting. 90 minutes per week.	Peer Mothers who have experienced a mental health condition	Deliver class	Trained, amount not specified	Intervention effectiveness, and evaluation Quantitative and qualitative questionnaire through a comparative study	Mother	Satisfaction, postpartum depression and clinical characteristics
Raine 2003 [77] UK	Mothers recruited at birth of infant. Recruitment method not reported.	**Sure Start Breastfeeding Support Project** Breastfeeding education and peer support	Group sessions in the community and telephone support. Weekly sessions plus telephone support.	Volunteer, Peer Mothers with breastfeeding experience	Provide breastfeeding peer support	2 hours x 12 weeks	Descriptive Qualitative interviews and research diaries	Mother, volunteers, project coordinators, midwives and health visitors	Experiences and perceived benefits of peer support
Rempel 2012 [95] Canada	Mothers recruited during pregnancy. Recruitment method not reported.	**Me? Breastfeed?** Breastfeeding education, peer support, and encouraging breastfeeding initiation and duration	Group sessions in a clinical setting. 2-hour one off session.	Volunteer breastfeeding buddies Mothers with a minimum of 6 months breastfeeding	Deliver class and provide peer support	18 hours	Intervention evaluation Questionnaire and qualitative interviews—quasi experimental design	Mothers and infant	Class evaluation, breastfeeding intentions up to two years of infant age, reasons for stopping breastfeeding, breastfeeding support, breastfeeding experience, duration of exclusive breastfeeding at one and six month postpartum.
Schwarz 2016 [53] USA	Mothers during prenatal period recruited via clinic waiting rooms.	Breastfeeding education and counselling	One to one in a clinical setting. One session.	Volunteer, Students Student with no clinical training	Educate and breastfeeding counselling	1 hour	Impact of intervention Pre post data collection	Mother	Intention to breastfeed. Knowledge of maternal health benefits of lactation
Shorey 2023 [56] Singapore	Parents recruited during pregnancy from two public health care institutions.	**SPA intervention** To support new parents across the perinatal period.	Access to a theory- and evidence-based psychoeducational mobile app SPA and emotional and peer support from a volunteer. 24 weeks gestation to 6 months postpartum	Peer volunteer Experienced mothers trained by a research team.	Provide emotional and peer support through the app.	Training amount not specified	Intervention effectiveness Randomised controlled trial	Parents	Postnatal depression, anxiety, parental bonding, parental self-efficacy, perceived social support and parenting satisfaction.
Spiby 2015 [96] UK	Disadvantaged mothers from 6 months pregnant to 6 weeks postpartum recruited via self-referral, clinical referral or organisation referral.	Support mental and emotional wellbeing and signposting to services, breastfeeding education, practical support and support attending appointments	Home visiting and telephone support. From 6 months pregnant to 6 weeks post partum.	Doula	Provide emotional support and signpost to services, labour support and breastfeeding support	Trained, amount not specified	Cost effectiveness and evaluation of services Routine data, survey and qualitative interviews conducted through a realistic evaluation perspective	Mothers, volunteers, project managers, project workers and midwives	Clinical and public health outcomes including, epidural use, rates of caesarean section, low birthweight, admission to neonatal unit, smoking and breastfeeding. Perceptions and experiences of service Costs of running a doula service and cost implications for the NHS were calculated
Spiby 2016 [97] UK							Descriptive Quantitative and qualitative questionnaire.	Volunteer	The motivation and experiences of volunteer doulas
Darwin 2017 [62] UK							Descriptive Questionnaire and qualitative interviews	Mother	The experiences of the women who used the service; the areas of impact and the nature of the relationship that may offer insights into how such outcomes occur.
Stone 2017 [98] UK	Parents of infants aged 8 weeks or above recruited via self-referral, clinical referral or organisation referral.	**A Good Start** Support mental and emotional wellbeing, signposting to services, and support attachment	Group sessions in the community. Duration not reported.	Volunteer Local parent who has previously received the intervention	Deliver baby massage class and signpost to services	Trained, amount not specified	Service evaluation Routine data and qualitative interviews	Parents (both), volunteers and staff	The extent to which the programme targets were met, using the ‘A Good Start Web’ outcome measurement tool,
Taggart 2000 [78] Australia	Mothers from birth of infant to 3 years. Targeted based on home address, being at risk, socially isolated, having poor health and/ or depression recruited via self-referral or clinical referral.	**Sutherland Family Network** Signposting to services, befriending and practical support	Home visiting. Duration not reported	Volunteer	Befriend and provide support	Trained, amount not specified	Descriptive Qualitative interviews	Mother, volunteers and project coordinators	Perceptions of experiences of intervention
Thomson 2012 [99] UK	Mothers of infants mostly aged under 1 month recruited via self-referral.	**National Breastfeeding Helplines** Breastfeeding education and support and signposting to services	Telephone support. One off session.	Volunteer Trained and registered breastfeeding counsellor	Provide emotional support, educate, signpost to services and breastfeeding support	Not reported	Service evaluation Structured quantitative and qualitative questionnaire telephone interview	Mother	How easy it was to access the helpline (number of call attempts); how many times they had used the service; perceptions of the opening hours; reason for calling the helpline; attitudes towards and impact of the help and support received; follow-up support options provided; overall satisfaction and recommendations for service development.
Wade 2009 [79] UK	Mothers recruited at birth of infant. Recruitment method not reported.	Breastfeeding education and peer support	Group sessions in the community, home visiting and telephone support. Duration not reported.	Peer supporter Mothers with breastfeeding experience	Provide peer support	Six sessions	Descriptive Qualitative focus groups	Mother	Benefits of interventions other than breastfeeding
Wong 2007 [54] Hong Kong	Mothers with an intention to breastfeed only from birth to infant aged 4 months old recruited from maternity ward.	Breastfeeding education	Telephone support. 7 sessions from period of birth to infant being aged 4 months.	Peer counselling volunteer Mothers with breastfeeding experience	Provide peer support and educate	20 hours	Intervention effectiveness Randomised Controlled Trial	Mother	Primary outcome: breastfeeding duration and exclusivity at 5 days, 3 months and 6 months post discharge. Additional outcomes: overall breastfeeding experience, evaluation of Peer Counselling (PC) support, breastfeeding knowledge, questions on breastfeeding plans and detailed questions on infant formula advertisements at 6 months post discharge.

Of the 66 evaluations included in the review, 21 studies [37–57] used quantitative methods only, 25 studies [58–82] used qualitative methods only and 20 studies [76, 83–101] used mixed methods to evaluate the intervention or explore the roles of volunteers. The most common data collection methods were qualitative interviews or focus groups (n = 33) and collecting questionnaire or survey data (n = 29).

A total of 48 studies collected data from the mother, with a further four studies [56, 89, 93, 98] collecting data from both parents and one study collecting data from fathers only [88]. 25 studies collected data from the volunteers [39, 47, 59, 61, 63–66, 71–74, 77, 78, 80, 85, 88, 89, 91, 93, 96–98, 100, 101] and 12 studies collected data from stakeholders involved in the intervention or service such as project managers or coordinators [77, 78, 80, 85, 89, 96, 98], healthcare staff [61, 65, 67, 75, 77, 96] or partner agencies [85, 93].

The most common aims of the identified research were to explore experiences or perceptions of those involved in interventions. These studies included exploring the experiences of mothers receiving (n = 21) [41, 58, 60, 62, 65, 69–71, 73, 74, 77, 78, 82, 84, 89, 92, 93, 96, 99–101] or volunteers delivering an intervention (n = 14) [63, 64, 70–74, 77, 78, 85, 89, 93, 96, 97], the perceived impact of receiving the intervention on outcomes (n = 8) [62, 77, 79, 88, 95, 98, 100, 101] and the motivations, benefits and factors relating to the sustainability (n = 5) of volunteering within interventions or services [65–68, 88]. Additionally, studies explored perceptions of the mother-volunteer relationship (n = 4) [59, 62, 67, 76] and volunteer-healthcare professional relationship (n = 2) [75, 86].

Further quantitative research aimed to determine intervention effectiveness through randomised controlled trials (n = 12) with six of which included primary outcomes relating to mental health outcomes [38, 39, 56, 63, 83, 102], five RCTs relating to breastfeeding rates [44, 45, 54, 94] and the primary outcome of one RCT was mother infant interactions [51].

There were 13 studies that collected data post intervention to explore the impact that the intervention had on change in outcomes relating to the aims of the intervention. Outcomes of interest included breastfeeding intention [53, 95], incidence [46, 96] and satisfaction [48], mental wellbeing [87] and specifically anxiety and depression. Additional outcomes explored related to birthing type [46, 96], mother-child relationship and social support [49, 85] and improved knowledge, confidence or skills in parenting [87, 98]. Remaining studies explored the feasibility (n = 5) [50, 52, 55, 90, 91], satisfaction (n = 5) [47, 88, 92, 99, 103], acceptability (n = 3) [41, 55, 90] and cost effectiveness (n = 3) [92, 96, 102] of an intervention.

#### Intervention design

The 54 interventions targeting parents or primary care givers of infants in their first 1001 days had a range of purposes and designs with some interventions including multiple components. The most common intervention purposes were providing support for the initiation or continuation of breastfeeding (n = 24) [43–46, 48, 52–55, 60, 61, 63, 67, 68, 70, 77, 79, 84, 88, 90, 94–96, 99], providing mental and emotional support to parents (n = 24)[39, 43, 46, 49, 51, 55, 56, 59, 60, 73, 75, 76, 83, 85–87, 89, 91, 93, 96, 98–100, 103] and signposting parents into services for further support (n = 15) [40, 43, 48, 49, 51, 70, 78, 82, 83, 89–91, 96, 98, 99]. The provision of peer support was commonly reported as an element, within 28 interventions [40, 41, 43–45, 48, 51, 52, 54, 56, 58, 61, 63, 65, 67, 70, 71, 76, 77, 79, 83, 84, 87, 88, 90, 94, 95, 103] predominantly in relation to breastfeeding support and supporting mothers with specific characteristics (i.e., first time mothers). Less common intervention components included teaching parent skills (n = 8) [38, 47, 49, 59, 68, 76, 83, 102], providing practical support (i.e., childcare) (n = 7) [38, 51, 68, 70, 78, 96, 100] and supporting parent-child attachment (n = 5) [51, 85, 89, 93, 98].

Interventions were most commonly delivered through home visiting (n = 29) [38, 45, 47, 49–51, 60, 64, 65, 68, 70–73, 76, 78, 79, 83, 85–87, 89, 90, 93, 94, 96, 100, 102], telephone support (n = 19) [39–41, 45, 47, 48, 54, 58, 63, 70–73, 77, 79, 94, 96, 99] and group sessions (n = 14) [46, 52, 65, 70, 76, 77, 79, 82, 84, 85, 88, 93, 95, 98] with some interventions using a combination of these approaches. Further delivery styles that were less commonly used included providing support in out of home settings such as clinical settings [53, 60, 61, 67, 75, 100] and via online or social media [44, 52, 56]. The duration and frequency of individual contacts or sessions within interventions were varied, ranging from a small number of interventions providing a one-off session only [88, 95, 99] to interventions that supported parents from pregnancy through to their infant being aged one or two years of age [70, 72, 82] (see Table 1 for individual intervention delivery approaches).

Mothers were the sole target of the majority of interventions (n = 47), with only one intervention targeting fathers only [88] and the remaining six interventions targeting both mothers and fathers [64, 65, 89, 93, 98]. Twenty-nine interventions were delivered to parents during their child’s infancy [39–41, 43, 44, 46–49, 51, 52, 54, 60, 61, 63, 64, 76–79, 84, 86, 90, 91, 94, 98, 99, 103], 21 interventions were delivered both during pregnancy and during their child’s infancy [38, 45, 50, 55, 56, 58, 65, 68, 70–73, 82, 83, 85, 87, 89, 93, 96, 100–102] and only four studies were delivered during pregnancy only [53, 59, 88, 95]. Of the interventions that targeted specific parents, common characteristics included those at risk of or living with a mental health condition [38–41, 51, 70, 76, 78, 85, 89], living in a deprived locality [45, 61, 93], being a young mother [59, 83, 102], having a migrant status [72, 73, 82, 86], being socially disadvantaged [45, 46, 49, 68, 96, 100] or socially isolated [73, 76, 78].

#### Volunteer characteristics

The titles given for volunteer roles varied across the identified interventions. Volunteers were most commonly referred to as ‘peers’ including ‘peer supporters’ [44, 48, 58, 61, 67, 70, 79, 90, 94], ‘peer volunteers’ [39–41, 43, 50, 51, 56, 63, 65, 77, 88, 103] and ‘peer support counsellors’ [54, 55, 84]. Eight interventions included individuals who worked in a voluntary capacity as ‘Doulas’ [46, 60, 68, 70, 73, 75, 96, 100] and less common titles were ‘mentors’ [59, 71, 91] and ‘befrienders’ [85, 89, 93]. Within almost all interventions (n = 51) it was specified that volunteers were provided with training; however, there was a large range in the amount of training provided. The most commonly reported duration of training was 4 hours or under (n = 11) [39–41, 43, 44, 50, 53, 59, 63, 88, 91] with the majority of papers stating that volunteers received less than 40 hours of training. Interventions that provided more than 40 hours of training included volunteers with titles of peer support counsellors [84], befrienders [85, 89] and Doulas [73, 74].

Where information about the background of volunteers was provided (n = 44), volunteers were most commonly parents themselves (n = 35) with the majority including volunteers who were mothers only (n = 30). One intervention [88] included volunteers who were fathers only and four interventions included volunteers who were mothers and fathers [38, 58, 93, 98]. Some volunteers were specifically included in interventions due to their personal experience of breastfeeding (n = 13) [45, 48, 52, 55, 61, 63, 70, 77, 79, 90, 94, 95, 99] or personal experiences of a mental health condition (n = 4) [39, 51, 70, 103]. Where volunteers were not specifically parents themselves, volunteers were described to be individuals with specific training, members of the local community and students.

Studies relating to 17 of the 55 interventions provided some demographic information for the volunteers. Thirteen studies reported the age of volunteers, with volunteers most commonly being aged in their 30s (n = 7) [47, 50, 59, 64, 66, 68, 96]. The remaining six studies reporting details of volunteer ages ranged from 20 to 60 years of age [44, 46, 70, 73, 85, 89]. Fewer studies (n = 10) provided details of volunteer’s ethnicity. Of those that did, six were conducted in the UK. Volunteers in three UK studies were described to be mostly white British [73, 85, 104], two studies included volunteers where approximately half of volunteers were white British and half of volunteers identified as either Black, Asian or Mixed Race [70, 72] and one study included volunteers where the largest proportion identified as Black Caribbean [89]. Three studies conducted in the USA reported ethnicity of volunteers with the majority of volunteers in studies identifying as White [47], as either White or African-American [46] or mostly African-American [59].The only Australian study to report ethnicity included volunteers who mostly identified as Caucasian [50].

#### Volunteer typologies

**Fig 3:**
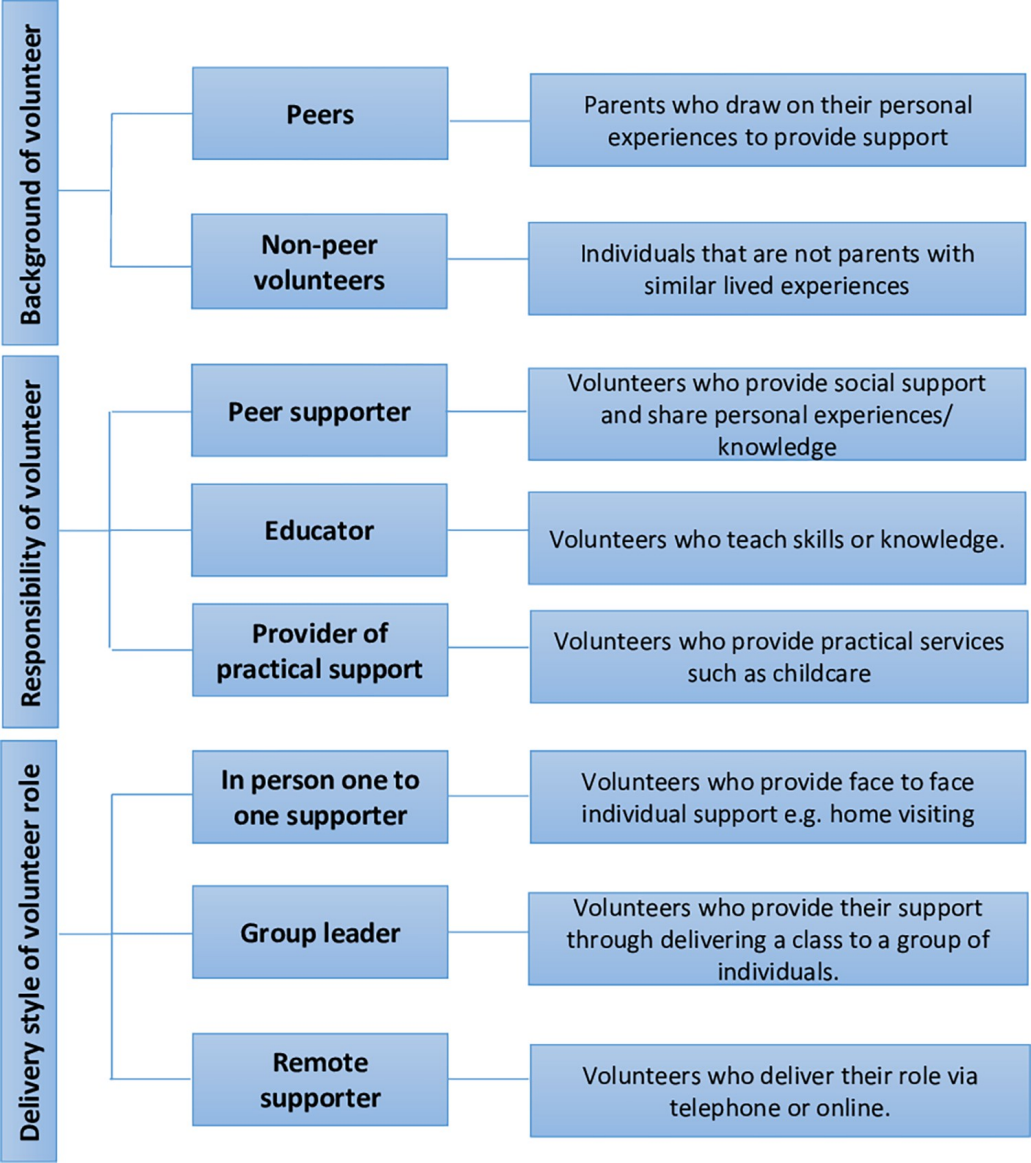
Typologies of volunteer roles within interventions for parents of infants in their first 1001 days.

Volunteers were categorised into typologies based on available information on the roles they take on and their personal backgrounds. Fig 3 presents and describes the three typologies of volunteer roles within interventions to support parents of infants in their first 1001 days.

Firstly, volunteers were classified based on their personal characteristics. Therefore, volunteers are categorised based on whether they are ‘peers’ who are individuals with similar characteristics or experiences as the service users or as ‘non-peer volunteers’, who are those that do not necessarily have personal similar experiences. Secondly, volunteers can be classified based on their responsibilities they take on as part of their role and volunteers who are ‘peer supporters’, ‘educators’ and ‘providers of practical support’. Volunteers can also be grouped based on the method they deliver the intervention and therefore could be grouped as either ‘in person one to one supporters’, as ‘group leaders’ or as ‘remote supporters’.

## Discussion

The evidence identified within this review has been explored to provide an understanding of the roles of volunteers in early years interventions, the characteristics of volunteers and the methods used to evaluate volunteer roles and the interventions they are involved in.

The review identified that individuals who take on volunteer roles within early years interventions are predominantly women and are often parents themselves. The use of peer-to-peer models has been identified to be a common approach within early years interventions, where parents are often paired with an individual with similar experiences. Peer support models are beneficial across interventions where trust and respect are required between recipients and those providing the support [105]. Peers have the ability to utilise their own personal experiences to reach out and engage with individuals through sharing their stories [106] with theoretical frameworks such as dynamic social impact theory and social cognitive theory explaining how shared experiences can encourage behaviour changes [107]. The high proportion of early years interventions designed to include volunteers who are peers of those receiving the intervention suggests there are perceived benefits to the mothers in having someone provide support who they can build trust with and learn from their similar personal experiences. Benefits to parents could include reduced stress, anxiety and depression and increased self-esteem and emotional wellbeing. These were identified as positive outcomes of peer support interventions in a previous review of early years interventions including volunteers by McLeish, Baker [9] which also identified peer support as a common role of volunteers.

As the majority of interventions targeted mothers, the use of peers likely explains why more women take on volunteer roles within early years interventions. However, due to many volunteers being parents of infants themselves, those volunteering within early years interventions are representing a younger demographic of volunteers than those often seen within other settings. Although volunteering roles are most likely to be taken up by women [15], the provision of volunteer roles within early years interventions could be an important setting for women who may have taken time out of work to care for their children to take up an active role in the community [108]. Further demographic data of volunteers was limited and therefore it was not possible to fully determine the ethnicity or socioeconomic status of individuals taking up volunteer roles within early years interventions. It is therefore unclear whether early years interventions are settings which attract and enable individuals of different ethnicities or socioeconomic status who often face more barriers to participating in volunteering to become involved [109]. Future research exploring the demographics of those volunteering within early years interventions would be beneficial to determine if the opportunity to volunteer and gain the potential benefits in this setting is available to all and, if not, could explore opportunities to make volunteering more accessible.

The evidence identified that volunteers are most commonly included within interventions to support the initiation and duration of breastfeeding and to support the mental and emotional wellbeing of mothers, reflecting two key public health priorities in the first 1001 days [1, 110, 111]. However, across the identified literature, volunteers were less likely to be involved in interventions to teach parenting skills, to provide practical support, or to support parent-child attachment despite being the focus of many interventions delivered by professionals or paid staff [6, 7, 112, 113]. This may suggest that volunteers are deemed more appropriate to be included in interventions where their previous experience (i.e., breastfeeding experience) or personal skills (i.e., ability empathise due to a peer experience) can be utilised compared to interventions such as those to teach parenting skills or support parent-child attachment where more specialist training may be required.

The review also highlighted that volunteers work within universally offered interventions as well as interventions targeting those deemed at higher risk (i.e. young mothers, individuals living in a deprived locality) with volunteers working within targeted interventions often having similar experiences (i.e. previous mental health condition) to those receiving the support. While volunteers are less commonly included in services that require specialist skills, the inclusion of volunteers within services for targeted groups suggest that volunteers could be well suited to working with ‘at risk’ groups either due to their peer experiences or due to their non statutory role.

In most interventions, volunteers were incorporated within a ‘Service Delivery Model’ in which volunteers delivered the intervention themselves as opposed to a ‘Support Role Model’ where volunteers would work alongside and support paid staff [114]. This suggests that, within early years interventions, volunteers are deemed appropriate to deliver a range of interventions themselves rather than providing additional support to paid staff or professionals. However, it has been previously highlighted that volunteer roles should not be seen as a cheaper replacement to a professional role and the qualities of volunteers as a non-professional role should be carefully considered during the decision to have the intervention delivered by volunteers in order for the intervention to be successful [9]. Given that volunteers are responsible for delivering important interventions with the potential to impact on the healthy development of children, there is justification for ongoing research into understanding if, how and why volunteer delivery models are suitable (or not) for all those involved, across the varied intervention designs.

Based on the information provided on how volunteers deliver their roles, a typology of delivery approaches was identified, including one to one in person support, group leader and remote support. This partly aligns with the model identified by McLeish, Baker [9] who also identified that volunteers were working both in a one on one capacity and as a group leader in interventions in the first four years of life. However, they also suggested a ‘Community champion’ approach, which was not evident in the identified literature, perhaps indicating it is a less common approach for interventions in the first 1001 days. Further, the evidence within this scoping review highlighted volunteers were often delivering part or all their support remotely. This typology can be considered within future research of volunteers within early years interventions to support the generation of more specific findings and recommendations for practice.

### Mapping of the evidence

This review has identified that half of the included publications on the use of volunteers have been published since the year 2015, highlighting an increased interest in recent years. Research was predominantly conducted in the UK, USA, Australia and Canada despite all countries classified as high income being eligible for inclusion. Data collated by the United Nations Volunteers (UNV) programme highlights that these are some of the countries with the highest rates of volunteering globally [18] and therefore could explain the larger proportion of identified studies.

We found that the purpose of evaluations, methods used and the outcomes or phenomena of interest to researchers varied. Qualitative research methods were most common across included studies, with the majority of the literature focusing on outcomes or experiences related to the parents and infants involved in the interventions. However, the evidence provided in this review highlighted that fewer studies have been conducted that aim to determine the effectiveness of interventions including volunteer roles and the effectiveness of volunteers delivering the interventions. Further randomised controlled trials of the different early years interventions designed to be delivered by volunteers would contribute to the understanding of if the inclusion of volunteer roles is an effective approach in achieving desired outcome. In addition, determining the effectiveness of the range of early years interventions involving volunteers could enable the comparison to early years interventions delivered by professionals or paid staff and explore if volunteer led interventions are as effective. Recent systematic reviews of parenting [6, 7], breastfeeding [115] and postnatal depression interventions [116] provide mixed findings with combined results showing little or no effect on outcomes, highlighting challenges in providing a benchmark to determine if volunteer led interventions are as effective.

Within the literature, and particularly research published over the last six years, there has been some interest in how volunteers experience their role or how they are impacted by the intervention themselves. Other areas of the health and social care sector where volunteers are commonly included such as palliative care [117] or dementia care [118] have explored the experiences of volunteers to understanding how volunteers perceive their roles and the impact the roles have on themselves as well as the intervention recipients. Further, the potential of volunteering as a public health intervention in itself has previously been explored with direct benefits to mental and physical wellbeing of the volunteers reported [13]. The suggested increase in research of the volunteer experience could imply that there is interest in determining whether the inclusion of volunteer roles within early years interventions is an appropriate approach to intervention delivery from the perspective of the volunteer and how the inclusion of volunteer roles in early years interventions could be a beneficial approach for both intervention recipient and volunteer.

While there is limited but increasing interest in the perceived impact on and experiences of volunteers highlighted in the early years evidence, there is also limited evidence on how the inclusion of volunteer roles in interventions impacts on the organisation delivering the intervention or staff and professionals linked to the intervention. The inclusion of volunteer roles has been found to have wider benefits than to those directly receiving the intervention in terms of increasing the capacity of the organisation and building links with the local community [11, 15] but has also been found to have negative impacts such as volunteers experiencing burnout [119, 120] and high volunteer attrition increasing burden on paid staff in organisations [17]. This review has identified only a small number of studies that have explored the experience of paid staff who work alongside or support volunteers. Exploring the perceptions of individuals who work within the organisation could provide insight into if volunteers are generating wider benefits to the organisation and considerations for how they could be best included within the organisation.

This review has provided an up to date understanding of the contribution of volunteers within early years interventions. Volunteers make a significant contribution to the provision of these important services and the healthy development of children in the early years. This review has provided consideration for those designing early years interventions of the potential roles volunteers can play within these services, along with the presentation of available evidence that can support their decision making. The typology presenting the varied roles can enable future research to explore these differences when evaluating the impact of volunteer led early years interventions. This can provide more specific guidance and recommendations for the successful delivery of the different early years interventions. Gaps in the literature in relation to understanding the effectiveness of the different volunteer led early years interventions and the impact of a volunteer delivery model on the volunteer and organisation have also been highlighted. This can provide guidance for where future research should be prioritised to support the understanding of the roles and impact of volunteers delivering early years interventions.

### Strengths and limitations

This review identified a large evidence base which has provided an understanding of the variety of volunteer roles within interventions delivered in the first 1001 days and has highlighted the characteristics of those taking up roles based on the most up to date literature. This information has supported the generation of a typology of volunteer roles which can be used to distinguish between the types of volunteer roles during future research. Additionally, the review has identified the extent of research previously conducted. An understanding of research previously conducted has identified where future research is needed based on gaps in the literature.

The findings presented in this review are predominantly from academic research sources and from service evaluations identified through searching grey literature sources. Therefore, the descriptions of volunteer roles and typologies created only reflects the contribution of volunteer roles from within the identified literature. It could therefore be beneficial to explore early years services currently being delivered to clarify that the findings of this review do reflect what is happening in practice. Secondly, this review can only provide a picture of the role of volunteers and extent of evaluation conducted within interventions for the first 1001 days of life rather than all early years interventions. In addition, all grey literature evidence sources searched during the review identified literature relating to interventions and services delivered in the UK. This could in part be due to the reviewer having more knowledge of UK organisations and therefore more UK websites searched and despite disabling location settings on Google, more UK webpages were displayed when using the search engine. Finally, while scoping review methods have allowed the mapping of the available evidence base, due to broad nature of the research question (and therefore evidence retrieved), it has not been possible to synthesise the findings of included studies and further systematic reviews would be beneficial in drawing conclusions of the findings of the current evidence base.

## Conclusion

This scoping review explored the evidence and enabled a typology to be created to distinguish the different backgrounds of volunteers, their roles and how they deliver their roles within early years interventions which can provide guidance of the capabilities of volunteers to those considering including volunteers in future early years interventions. In addition, the development of a typology can support the description and classification of volunteer roles across a broad spectrum of early years interventions.

The literature to date has mostly focused on the experiences of those receiving the intervention and the impact the intervention had on health and wellbeing outcomes of the parent or their infant. The review has highlighted that less research has explored the implications of including volunteer roles on the organisation hosting, or the volunteers themselves. In addition, fewer studies have explored the effectiveness of early years interventions delivered by volunteers. Further research exploring the effectiveness of early years interventions that include volunteer roles and effectiveness of volunteers delivering interventions on different outcomes and research exploring the wider impact on the organisation would help provide a clearer picture of the impact of early years interventions being delivered by volunteers.

## Supporting information

S1 Checklist PRISMA-ScR checklist. (DOCX)

S1 Text Search strategy. (DOCX)
