# Supplementary material for: Use of volunteers in early years interventions for parents: A scoping review of roles and the extent of evaluation research in this area
Source: PLoS One. 2024 Sep 26;19(9):e0305551. doi: 10.1371/journal.pone.0305551 (PMC11426509; doi:10.1371/journal.pone.0305551)
Supplement: S1 Text — (DOCX) [file pone.0305551.s002.docx]

**Ovid MEDLINE(R) and Epub Ahead of Print, In-Process, In-Data-Review & Other Non-Indexed Citations and Daily <1946 to May 21, 2021>**

1 Early years.ti,ab. 4178

2 First 1000 days.ti,ab. 247

3 Early childhood.ti,ab. 27806

4 Infant/ 812635

5 Infant.ti,ab. 173835

6 Infancy.ti,ab. 53872

7 Baby.ti,ab. 39633

8 Babies.ti,ab. 38403

9 Toddler*.ti,ab. 11955

10 First years.ti,ab. 4105

11 P*diatric.ti,ab. 340606

12 Antenatal.ti,ab. 37803

13 Postnatal.ti,ab. 108402

14 Neonatal.ti,ab. 214450

15 New-born.ti,ab. 4062

16 Mother*.ti,ab. 231255

17 Parent*.ti,ab. 434616

18 Carer*.ti,ab. 15695

19 Primary carer*.ti,ab. 265

20 Family.ti,ab. 828079

21 Families.ti,ab. 254832

22 Maternity.ti,ab. 22238

23 Maternal.ti,ab. 267225

24 Father.ti,ab. 24824

25 Paternal.ti,ab. 23729

26 Pregnancy.ti,ab. 399590

27 Pregnancy/ 901631

28 Pregnant.ti,ab. 190638

29 Expectant mothers.ti,ab. 1091

30 Volunteer*.ti,ab. 199528

31 Volunteers/ 10009

32 Unpaid worker*.ti,ab. 16

33 Unpaid staff.ti,ab. 5

34 Lay support.ti,ab. 57

35 Peer support*.ti,ab. 5030

36 Peer to peer.ti,ab. 1557

37 Parent to parent.ti,ab. 463

38 Community champion*.ti,ab. 28

39 Befriender*.ti,ab. 48

40 Peer educator*.ti,ab. 758

41 peer counsel*.ti,ab. 573

42 Peer.ti,ab. 74548

43 Mentor*.ti,ab. 17417

44 Charity worker*.ti,ab. 9

45 Voluntary worker*.ti,ab. 74

46 Voluntary staff.ti,ab. 17

47 Doula*.ti,ab. 385

48 Intervention.ti,ab. 650021

49 Service*.ti,ab. 547332

50 Support.ti,ab. 1059446

51 Program*.ti,ab. 925916

52 Parenting.ti,ab. 20313

53 Parenting/ 17848

54 Parenting skills.ti,ab. 875

55 Parent* confidence.ti,ab. 308

56 Parent* satisfaction.ti,ab. 1866

57 Wellbeing.ti,ab. 18869

58 Emotional wellbeing.ti,ab. 859

59 Mental wellbeing.ti,ab. 930

60 Psychological wellbeing.ti,ab. 1570

61 Mental Health/ 43622

62 Attachment.ti,ab. 108918

63 Relationship.ti,ab. 1076720

64 Breast Feeding/ 39160

65 Breastfeed*.ti,ab. 28685

66 Social development.ti,ab. 3282

67 Emotional development.ti,ab. 1702

68 Behavi* development.ti,ab. 2116

69 Child Development/ 47699

70 1 or 2 or 3 or 4 or 5 or 6 or 7 or 8 or 9 or 10 or 11 or 12 or 13 or 14 or 15 or 16 or 17 or 18 or 19 or 20 or 21 or 22 or 23 or 24 or 25 or 26 or 27 or 28 or 29 **3558967**

71 30 or 31 or 32 or 33 or 34 or 35 or 36 or 37 or 38 or 39 or 40 or 41 or 42 or 43 or 44 or 45 or 46 or 47 **294107**

72 48 or 49 or 50 or 51 **2754367**

73 52 or 53 or 54 or 55 or 56 or 57 or 58 or 59 or 60 or 61 or 62 or 63 or 64 or 65 or 66 or 67 or 68 or 69 **1348090**

74 70 and 71 and 72 and 73 **3343**

75 limit 74 to english language **3285**

76 limit 75 to yr="2000 -Current" **2950**

**APA PsycInfo <1987 to May Week 3 2021>**

1 Early years.ti,ab. 4117

2 First 1000 days.ti,ab. 28

3 Early childhood.ti,ab. 23952

4 Infant.ti,ab. 36258

5 Infancy.ti,ab. 14751

6 Baby.ti,ab. 10397

7 Babies.ti,ab. 5312

8 Toddler*.ti,ab. 9290

9 First years.ti,ab. 1344

10 P*diatric.ti,ab. 31401

11 Antenatal.ti,ab. 3613

12 Postnatal.ti,ab. 19045

13 Neonatal.ti,ab. 14792

14 New-born.ti,ab. 192

15 Mother*.ti,ab. 106226

16 Parent*.ti,ab. 242896

17 Carer*.ti,ab. 10554

18 Primary carer*.ti,ab. 213

19 Family.ti,ab. 271587

20 Families.ti,ab. 129804

21 Maternity.ti,ab. 3001

22 Maternal.ti,ab. 52143

23 Father.ti,ab. 20090

24 Paternal.ti,ab. 9342

25 Pregnancy/ 21376

26 Pregnancy.ti,ab. 34710

27 Pregnant.ti,ab. 17782

28 Expectant mothers.ti,ab. 311

29 Volunteer*.ti,ab. 34843

30 exp Volunteers/ 4636

31 Unpaid worker*.ti,ab. 16

32 Unpaid staff.ti,ab. 6

33 Lay support.ti,ab. 24

34 Peer support*.ti,ab. 4556

35 Peer to peer.ti,ab. 1571

36 Parent to parent.ti,ab. 744

37 Community champion*.ti,ab. 10

38 Befriender*.ti,ab. 71

39 Peer educator*.ti,ab. 462

40 peer counsel*.ti,ab. 493

41 Peer.ti,ab. 61927

42 Mentor*.ti,ab. 17581

43 Charity worker*.ti,ab. 5

44 Voluntary worker*.ti,ab. 41

45 Voluntary staff.ti,ab. 7

46 Doula*.ti,ab. 128

47 Intervention.ti,ab. 236051

48 Service*.ti,ab. 261987

49 Support.ti,ab. 457322

50 Program*.ti,ab. 349991

51 Parenting.ti,ab. 39213

52 Parenting/ 11475

53 Parenting skills.ti,ab. 1672

54 Parenting Skills/ 2752

55 Parent* confidence.ti,ab. 287

56 Parent* satisfaction.ti,ab. 1243

57 Wellbeing.ti,ab. 14733

58 Emotional wellbeing.ti,ab. 728

59 Mental wellbeing.ti,ab. 596

60 Psychological wellbeing.ti,ab. 1507

61 Mental Health/ 66143

62 Attachment.ti,ab. 38536

63 Relationship.ti,ab. 461716

64 Breastfeed*.ti,ab. 4207

65 Social development.ti,ab. 5191

66 Emotional development.ti,ab. 4295

67 Behavi* development.ti,ab. 2140

68 Childhood Development/ 63659

69 Child development.ti,ab. 9852

70 1 or 2 or 3 or 4 or 5 or 6 or 7 or 8 or 9 or 10 or 11 or 12 or 13 or 14 or 15 or 16 or 17 or 18 or 19 or 20 or 21 or 22 or 23 or 24 or 25 or 26 or 27 or 28 **657724**

71 29 or 30 or 31 or 32 or 33 or 34 or 35 or 36 or 37 or 38 or 39 or 40 or 41 or 42 or 43 or 44 or 45 or 46 **113003**

72 47 or 48 or 49 or 50 **1036153**

73 51 or 52 or 53 or 54 or 55 or 56 or 57 or 58 or 59 or 60 or 61 or 62 or 63 or 64 or 65 or 66 or 67 or 68 or 69 **646444**

74 70 and 71 and 72 and 73 **5008**

75 limit 74 to english language **4741**

76 limit 75 to yr="2000 -Current" **4186**

77 limit 76 to journal article **2290**

**Embase <1996 to 2021 Week 20>**

1 Early years.ti,ab. 4538

2 First 1000 days.ti,ab. 346

3 Early childhood.ti,ab. 30925

4 infant/ 458395

5 Infant.ti,ab. 153281

6 Infancy.ti,ab. 51911

7 Baby.ti,ab. 45908

8 Babies.ti,ab. 42280

9 Toddler*.ti,ab. 14319

10 First years.ti,ab. 5235

11 P*diatric.ti,ab. 486288

12 Antenatal.ti,ab. 46437

13 Postnatal.ti,ab. 110466

14 Neonatal.ti,ab. 225124

15 New-born.ti,ab. 3574

16 Mother*.ti,ab. 241103

17 Parent*.ti,ab. 456571

18 Carer*.ti,ab. 22821

19 Primary carer*.ti,ab. 359

20 Primary carer*.ti,ab. 359

21 Family.ti,ab. 925292

22 Families.ti,ab. 274071

23 Maternity.ti,ab. 23713

24 Maternal.ti,ab. 290243

25 Father.ti,ab. 28551

26 Paternal.ti,ab. 25677

27 Pregnancy.ti,ab. 410727

28 pregnancy/ 387871

29 Pregnant.ti,ab. 206730

30 Expectant mothers.ti,ab. 1034

31 Volunteer*.ti,ab. 217765

32 volunteer/ 52332

33 Unpaid worker*.ti,ab. 13

34 Unpaid staff.ti,ab. 3

35 Lay support.ti,ab. 61

36 Peer support*.ti,ab. 6924

37 Peer to peer.ti,ab. 2211

38 Parent to parent.ti,ab. 516

39 Community champion*.ti,ab. 45

40 Befriender*.ti,ab. 45

41 Peer educator*.ti,ab. 923

42 peer counsel*.ti,ab. 610

43 Peer.ti,ab. 84009

44 Mentor*.ti,ab. 22234

45 Charity worker*.ti,ab. 14

46 Voluntary worker*.ti,ab. 43

47 Voluntary staff.ti,ab. 23

48 Doula*.ti,ab. 369

49 Intervention.ti,ab. 909058

50 Service*.ti,ab. 617664

51 Support.ti,ab. 1213985

52 Program*.ti,ab. 1060408

53 Parenting.ti,ab. 22070

54 Parenting skills.ti,ab. 1004

55 Parent* confidence.ti,ab. 399

56 Parent* satisfaction.ti,ab. 2441

57 Wellbeing.ti,ab. 28400

58 Emotional wellbeing.ti,ab. 1660

59 Mental wellbeing.ti,ab. 1376

60 Psychological wellbeing.ti,ab. 2574

61 mental health/ 142237

62 Attachment.ti,ab. 100596

63 Relationship.ti,ab. 1165725

64 breast feeding/ 46784

65 Breastfeed*.ti,ab. 33734

66 Social development.ti,ab. 3324

67 Emotional development.ti,ab. 1983

68 Behavi* development.ti,ab. 2398

69 child development/ 34682

70 1 or 2 or 3 or 4 or 5 or 6 or 7 or 8 or 9 or 10 or 11 or 12 or 13 or 14 or 15 or 16 or 17 or 18 or 19 or 20 or 21 or 22 or 23 or 24 or 25 or 26 or 27 or 28 or 29 or 30 **3122090**

71 31 or 32 or 33 or 34 or 35 or 36 or 37 or 38 or 39 or 40 or 41 or 42 or 43 or 44 or 45 or 46 or 47 or 48 **326558**

72 49 or 50 or 51 or 52 **3239809**

73 53 or 54 or 55 or 56 or 57 or 58 or 59 or 60 or 61 or 62 or 63 or 64 or 65 or 66 or 67 or 68 or 69 **1508593**

74 70 and 71 and 72 and 73 **4578**

75 limit 74 to english language **4500**

76 limit 75 to yr="2000 -Current" **4366**

77 limit 76 to embase **1738**

**HMIC Health Management Information Consortium <1979 to March 2021>**

1 Early years.ti,ab. 340

2 First 1000 days.ti,ab. 2

3 Early childhood.ti,ab. 321

4 Infant.ti,ab. 1843

5 Infancy.ti,ab. 463

6 Baby.ti,ab. 1365

7 Babies.ti,ab. 1400

8 Toddler*.ti,ab. 124

9 First years.ti,ab. 99

10 P*diatric.ti,ab. 2059

11 Antenatal.ti,ab. 1239

12 Postnatal.ti,ab. 829

13 Neonatal.ti,ab. 1377

14 New-born.ti,ab. 39

15 Mother*.ti,ab. 4159

16 Parent*.ti,ab. 8939

17 Carer*.ti,ab. 7321

18 Primary carer*.ti,ab. 36

19 Family.ti,ab. 13018

20 Families.ti,ab. 7798

21 Maternity.ti,ab. 2707

22 Maternal.ti,ab. 2348

23 Father.ti,ab. 313

24 Paternal.ti,ab. 168

25 Pregnancy/ 2273

26 Pregnancy.ti,ab. 3524

27 Pregnant.ti,ab. 1565

28 Expectant mothers.ti,ab. 58

29 Volunteer*.ti,ab. 1830

30 exp Volunteers/ 384

31 Unpaid worker*.ti,ab. 6

32 Unpaid staff.ti,ab. 0

33 Lay support.ti,ab. 7

34 Peer support*.ti,ab. 303

35 Peer to peer.ti,ab. 19

36 Parent to parent.ti,ab. 14

37 Community champion*.ti,ab. 3

38 Befriender*.ti,ab. 25

39 Peer educator*.ti,ab. 32

40 peer counsel*.ti,ab. 10

41 Peer.ti,ab. 2241

42 Mentor*.ti,ab. 705

43 Charity worker*.ti,ab. 1

44 Voluntary worker*.ti,ab. 36

45 Voluntary staff.ti,ab. 9

46 Doula*.ti,ab. 4

47 Intervention.ti,ab. 10622

48 Service*.ti,ab. 98131

49 Support.ti,ab. 30705

50 Program*.ti,ab. 28457

51 Parenting.ti,ab. 671

52 Parenting/ 189

53 Parenting skills.ti,ab. 61

54 Parent* confidence.ti,ab. 10

55 Parent* satisfaction.ti,ab. 66

56 Wellbeing.ti,ab. 2664

57 Emotional wellbeing.ti,ab. 82

58 Mental wellbeing.ti,ab. 165

59 Psychological wellbeing.ti,ab. 139

60 Mental Health/ 6561

61 Attachment.ti,ab. 419

62 Relationship.ti,ab. 11593

63 Breastfeed*.ti,ab. 658

64 Social development.ti,ab. 90

65 Emotional development.ti,ab. 46

66 Behavi* development.ti,ab. 19

67 Child development.ti,ab. 267

68 1 or 2 or 3 or 4 or 5 or 6 or 7 or 8 or 9 or 10 or 11 or 12 or 13 or 14 or 15 or 16 or 17 or 18 or 19 or 20 or 21 or 22 or 23 or 24 or 25 or 26 or 27 or 28 **42256**

69 29 or 30 or 31 or 32 or 33 or 34 or 35 or 36 or 37 or 38 or 39 or 40 or 41 or 42 or 43 or 44 or 45 or 46 **4878**

70 47 or 48 or 49 or 50 **138160**

71 51 or 52 or 53 or 54 or 55 or 56 or 57 or 58 or 59 or 60 or 61 or 62 or 63 or 64 or 65 or 66 or 67 **21666**

72 68 and 69 and 70 and 71 **115**

73 limit 72 to english **98**

**Applied Social Sciences Index & Abstracts (ASSIA)**

Searched for:ab("Early years" OR "First 1000 days" OR "Early childhood" OR Infant OR Infancy OR Baby OR Babies OR Toddler* OR "First years" OR Antenatal OR Postnatal OR New*born OR Mother* OR Parent* OR Carer* OR "Primary carer*" OR Maternity OR Maternal OR Father OR Paternal OR Pregnancy OR Pregnant OR "Expectant mothers" OR Family OR Families) AND ab(Volunteer* OR "Unpaid worker*" OR "Unpaid staff" OR "Peer support*" OR "Community champion*" OR Befriender* OR "Peer educator*" OR Peer OR "Peer counsel*" OR "Parent to parent" OR Mentor* OR "Charity worker*" OR "Voluntary worker*" OR "Voluntary staff" OR Doula*) AND ab(Intervention OR Service* OR Support OR Program*) AND ab(Parenting OR "Parenting skills" OR "Parent* confidence" OR "Parent* satisfaction" OR Wellbeing OR "Emotional wellbeing" OR "Mental wellbeing" OR "Psychological wellbeing" OR "Mental health" OR Attachment OR Relationship OR "Social development" OR "Emotional development" OR "Behavi* development" OR "Child development" OR breastfeed*) AND stype.exact("Scholarly Journals") AND la.exact("English") AND pd(>19991231)

Limited by:

Date: After 31 December 1999

Source type:Scholarly Journals

Language:English

Databases:

Applied Social Sciences Index & Abstracts (ASSIA)

Total: **2483**

**Web of Science**

(((TS=(“Early years” OR “First 1001 days” OR “First 1000 days” OR “Early childhood” OR Infant OR Infancy OR Baby OR Babies OR Toddler* OR “First years” OR Antenatal OR Postnatal OR New*born OR Mother* OR Parent* OR Carer* OR “Primary carer*” OR Maternity OR Maternal OR Father OR Paternal OR Pregnancy OR Pregnant OR “Expectant mothers” OR Family OR Families )) AND TS=(Volunteer* OR “Unpaid worker*” OR “Unpaid staff” OR “Peer support*” OR “Community champion*” OR Befriender* OR “Peer educator*” OR Peer OR “Peer counsel*” OR “Parent to parent” OR Mentor* OR “Charity worker*” OR “Voluntary worker*” OR “Voluntary staff” OR Doula*)) AND TS=(Intervention OR Service* OR Support OR Program* )) AND TS=(Parenting OR “Parenting skills” OR “Parent* confidence” OR “Parent* satisfaction” OR Wellbeing OR “Emotional wellbeing” OR “Mental wellbeing” OR “Psychological wellbeing” OR “Mental health” OR Attachment OR Relationship OR “Social development” OR “Emotional development” OR “Behavi* development” OR “Child development” or breastfeed*) and English (Languages) and Article (Document Types)

Total = **5487**

Web of Science

(((TS=(“Early years” OR “First 1001 days” OR “First 1000 days” OR “Early childhood” OR Infant OR Infancy OR Baby OR Babies OR Toddler* OR “First years” OR Antenatal OR Postnatal OR New*born OR Mother* OR Parent* OR Carer* OR “Primary carer*” OR Maternity OR Maternal OR Father OR Paternal OR Pregnancy OR Pregnant OR “Expectant mothers” OR Family OR Families )) AND TS=(Volunteer* OR “Unpaid worker*” OR “Unpaid staff” OR “Peer support*” OR “Community champion*” OR Befriender* OR “Peer educator*” OR Peer OR “Peer counsel*” OR “Parent to parent” OR Mentor* OR “Charity worker*” OR “Voluntary worker*” OR “Voluntary staff” OR Doula*)) AND TS=(Intervention OR Service* OR Support OR Program* )) AND TS=(Parenting OR “Parenting skills” OR “Parent* confidence” OR “Parent* satisfaction” OR Wellbeing OR “Emotional wellbeing” OR “Mental wellbeing” OR “Psychological wellbeing” OR “Mental health” OR Attachment OR Relationship OR “Social development” OR “Emotional development” OR “Behavi* development” OR “Child development” or breastfeed*) and English (Languages) and Article (Document Types)

Total = 5487
